# Oil-in-water emulsion loaded with optimized antioxidant blend improved the shelf-life of trout (*Oncorhynchus mykiss*) fillets: a study with simplex-centroid design

**DOI:** 10.1038/s41598-024-55308-x

**Published:** 2024-02-27

**Authors:** Luiz Torres Neto, Maria Lucia G. Monteiro, Bruno D. da Silva, Diego Galvan, Carlos A. Conte-Junior

**Affiliations:** 1 Center for Food Analysis of Technological Development Support Laboratory (NAL; LADETEC), Institute of Chemistry, Federal University of Rio de Janeiro (UFRJ), Avenida Horácio Macedo, Bloco C, 1281 – Cidade Universitária, Rio de Janeiro, RJ 21941-598 Brazil; 2 Graduate Program in Food Science (PPGCAL), Institute of Chemistry of Federal University of Rio de Janeiro (IQ; UFRJ), Cidade Universitária, Rio de Janeiro, RJ 21941-909 Brazil; 3 Graduate Program in Veterinary Hygiene (PPGHV), Faculty of Veterinary Medicine, Fluminense Federal University (UFF), Vital Brazil Filho, Niterói, RJ 24220-000 Brazil; 4 Institute of Chemistry, Federal University of Santa Catarina (UFSC), Florianopolis, SC 88040-900 Brazil; 5 Graduate Program in Sanitary Surveillance (PPGVS), National Institute of Health Quality Control of Oswaldo Cruz Foundation (INCQS; FIOCRUZ), Rio de Janeiro, RJ 21040-900 Brazil

**Keywords:** Antioxidant, Volatile oils, Natural compounds, Refrigerated storage, Desirability function, Chemistry, Natural products

## Abstract

This study aimed to obtain optimized mixture with three essential oils (EOs) for maximum antioxidant activity through the augmented simplex-centroid mixture design and evaluate the effect of this optimized blend on total aerobic psychrotrophic count (TAPC), lipid and protein oxidation, instrumental color parameters and texture profile in rainbow trout fillets at refrigerated storage for nine days. Considering the DPPH and FRAP assays, the ideal EO blend was 66% lemongrass and 34% oregano. During refrigerated storage, this blend at 2000 ppm was equally effective as BHT (100 ppm) (*p* > 0.05), mitigating the discoloration (a* and b*), lipid, and protein oxidation in 38.83%, 12.95%, 76.13%, and 35.13%, respectively, besides shows greater effectiveness for preserving texture changes (*p* < 0.05) and extending the shelf life in 13 h. The lemongrass + oregano EO blend reveals a promising natural alternative to enhance the quality of rainbow trout fillets under refrigerated storage. Furthermore, the multiresponse optimization showed to be a strong ally in enabling the use of these EOs by food industries.

## Introduction

Rainbow trout (*Oncorhynchus mykiss*) is a rich source of nutrients like omega-3 (ω-3) series of polyunsaturated fatty acids, proteins, minerals, and vitamins, besides being one of the freshwater fish species with high importance for boosting the global fish trade due to the high acceptability and versatility to produce several fish products^1^. Furthermore, rainbow trout is among 15 major aquaculture species produced worldwide, achieving 739.50 thousand tonnes in 2020, representing 1.5% of the global aquaculture production^1^. Indeed, the global trout market is valued at US$ 4.2 billion, with growth projected to reach the US$ 7 billion mark by 2033 and a Compound Annual Growth Rate of 5.2%^2^. Furthermore, trout production in Brazil is also growing, with regions of the country reaching the mark of 2 thousand tons per year^3,4^. However, its high perishability is a limiting factor, rapidly reducing its shelf life by causing sensory changes and thus early consumer rejection and discarding^5–7^. This is attributed to its dark muscle composition and biochemistry, which presents high proteases activity, high content of lipids containing a higher unsaturated than satured fatty acids, and high content of myoglobin with low hemoglobin stability, favoring an accelerated microbial growth and lipid and protein oxidations^8–10^.

According to the FAO, public health risks and high yearly waste (30–35%) from high fish perishability represent the main barriers to sustainably developing the global fish production chain. Therefore, this reputable organization has encouraged studies for new conservation methods to assure the quality and safety towards fish consumption, meeting the Sustainable Development Goal (SDG), which aims to increase the supply following the growing demand for fish and halving Food Loss and Waste (FLW)^1^.

Among several emerging preservation food technologies studied (e.g., cold plasma, irradiation, UV-C light, pulsed electric fields, edible coatings, etc.), EOs have aroused wide attention due mainly to their cost-effectiveness and sustainable claim. EOs have a broad composition conferring them great antibacterialand antioxidant activities^11^. Furthermore, these oils are classified as generally recognized as safe (GRAS) and are considered a eco-friendly plant-based alternatives against pathogens and oxidative degradation in food^12–14^. However, the increase of EO concentrations are required to achieve bacterial growth control or antioxidant effectiveness, generating a negative sensory impact in food products^15,16^. In response to these drawbacks, the synergistic or additive mixture of different EOs has been proposed to achieve greater activity and spectrum of action with lower concentrations. Therefore, developing EO blends can also be a promising alternative in reducing the sensorial influence of EOs in food matrices, mainly fish.

The Mixture Designs (MDs) are emerging techniques for the development of optimized EO blends. The MDs allow the mixture of three components (e.g., EOs), which can be evaluated in different proportions. Furthermore, this experimental design enables the optimization of single or multiple responses simultaneously through the desirability functions^17^. Some studies have already used this technique to optimize the in vitro antioxidant^13,18,19^ and antimicrobial activities of EO blends against different microorganisms^12,20^. However, none of these studies evaluated the efficiency of these optimized EO blends in food matrices, indicating a gap to be filled in the literature.

The *Origanum vulgare* (oregano; ORE), *Thymus vulgaris* (thyme; THY), and *Cymbopogon citratus* (lemongrass; LG) EOs have a great single antioxidant activity already documented in the literature^21–23^. Furthermore, during 2021 and 2022, these OEs moved more than US$ 260 million in exports, mainly from countries such as China, India, Germany, and the Netherlands^24–26^. Most studies on natural antioxidant treatment to enhance the shelf life of fish, such as Bitalebi et al.^27^, have investigated fruit extracts, lacking studies about refrigerated stored fish quality added of EOs, especially EO mixtures. Recently, our research group published promising results regarding the simultaneous antimicrobial activity of a blend with these three EOs against *Escherichia coli*, *Staphylococcus aureus*, and *Salmonella enterica* serotype Enteritidis^16^*.* However, no study has proposed to develop an optimized antioxidant blend with ORE, THY, and LG considering distinct antioxidant mechanisms such as hydrogen atom transfer (HAT) and single electron transfer (SET) and further application in refrigerated stored trout fillets for extending its shelf life and mitigating its oxidative degradation.

Based on this, our study aimed to obtain an optimized formulation containing ORE, THY, and LG EOs for maximum antioxidant activity according to SET and HAT mechanisms through an augmented simplex-centroid mixture design and evaluate the potential of three different concentrations (100, 1000 and 2000 ppm) of the optimized blend in enhancing the shelf life and preventing the oxidative degradation in trout (*Oncorhynchus mykiss*) fillets stored at 4 °C for 9 days.

## Material and methods

### Material

The Tween 80 used as a surfactant was purchased from Rei-Sol (Rio de Janeiro, Brazil), the 2,2-diphenyl-1-picrylhydrazyl (DPPH), 2,4,6-Tris(2-pyridyl)-s-triazine (TPTZ) and (S)-trolox methyl ether (Trolox) from Sigma-Aldrich (São Paulo, Brazil), and butylhydroxytoluene (BHT) from Orion (Rio de Janeiro, Brazil). All three EOs were purchase from Quinari® (Ponta Grossa, PR, Brazil). The composition of the EOs was previously characterized through gas chromatography with mass spectrometry (GC–MS) and flame-ionization detection (FID), which can be seen in our previous study^16^.

### Mixture design

The augmented simplex-centroid design was used to achieve and optimize the simultaneous antioxidant effect of three EOs (ORE, THY, and LG)^17,28,29^. For this study, twelve experiments were performed, including three replications at the center point (experiments 7, 8 and 9; Table 1).

**Table 1:** Simplex-centroid design, DPPH radical reducing, and ferric ion reducing values of single and blended essential oils.

Runs	Mixture*			DPPH (% of inhibition)	FRAP (μmol TROLOX equivalent/g)
	ORE	THY	LG		
1	1	0	0	75.07 ± 0.20	98.42 ± 2.39
2	0	1	0	77.94 ± 0.20	46.57 ± 0.19
3	0	0	1	96.43 ± 0.30	17.46 ± 0.91
4	0.5	0.5	0	75.89 ± 0.49	60.25 ± 2.25
5	0.5	0	0.5	79.54 ± 1.17	51.72 ± 1.07
6	0	0.5	0.5	85.65 ± 1.05	30.99 ± 0.51
7	0.33	0.33	0.33	80.90 ± 0.56	51.63 ± 0.79
8	0.33	0.33	0.33	77.89 ± 0.70	49.40 ± 1.23
9	0.33	0.33	0.33	79.53 ± 0.74	45.88 ± 0.06
10	0.67	0.17	0.17	77.92 ± 0.09	63.37 ± 0.93
11	0.17	0.67	0.17	78.94 ± 0.12	48.12 ± 1.24
12	0.17	0.17	0.67	84.91 ± 0.79	32.33 ± 0.70

*Proportion of each oregano (ORE), thyme (THY), and lemongrass (LG) essential oil (EO).

The quality of linear, quadratic, and special cubic least-squares regression models was verified based on ANOVA. After this preliminary step, the quadratic model showed better quality based on R^2^, R^2^_adj._, lack-of-fit, and *p*-value (See Table 2). This model allows obtaining the dependent variable's responses in the function of the independent ones, as shown in Eq. (1).

1 $${\text{Y}} = \upalpha _{1} {\text{X}}_{1} + \upalpha _{2} {\text{X}}_{2} + \upalpha _{3} {\text{X}}_{3} + \upalpha _{12} {\text{X}}_{12} + \upalpha _{13} {\text{X}}_{13} + \upalpha _{23} {\text{X}}_{23} +\upvarepsilon$$

where: Y is the DPPH or FRAP assay (Section “Antioxidant assays”); the coefficients for linear terms are α_1_, α_2_, α_3_, binary terms are α_12_, α_13_, α_23_, and independent variables (ORE, THY, and LG) are X_1_, X_2,_ and X_3_, and error term is ε.

**Table 2:** ANOVA analysis on the quadratic model for DPPH and FRAP assays.

	Sum of squares	Degrees of freedom	Mean square	*F*-value	*P*-value	*R*^2^	*R*^2^_adj._*
DPPH							
Model	364.2986	5	72.85971	48.99955	0.000087		
Total error	8.9217	6	1.48695				
Lack of fit	4.3699	4	1.09247	0.48002	0.760090	0.9761	0.9562
Pure error	4.5518	2	2.27589				
Total adjusted	373.2202	11	33.92911				
FRAP							
Model	4335.540	5	867.1079	83.54769	0.000018		
Total error	62.272	6	10.3786				
Lack of fit	45.449	4	11.3621	1.35079	0.467327	0.9858	0.9740
Pure error	16.823	2	8.4115				
Total adjusted	4397.811	11	399.8010				

^*^R^2^_adj_: R^2^ adjusted.

#### Antioxidant assays

##### DPPH free radical scavenging activity

The reduction capacity of EOs and their mixtures front 2,2-diphenyl-1-picrylhydrazyl (DPPH) radical was determined according to Rufino et al.^30^ with modifications^18^. For that, 100 μL of EOs and their mixtures (from 240 mg/mL in methanol) were individually added to 3.9 mL of DPPH methanol solution (0.06 mM), followed by homogenization and left in the dark for 90 min. A control without the presence of EOs was also performed. After the reaction period, the absorbance of the samples was read in a spectrophotometer (UV–VIS 1900i, Shimadzu, Tokyo, TKY, Japan) at 515 nm. The % of DPPH radical inhibition rate was determined applying the absorbance values (A) in the Eq. (2):

2 $${\text{Inhibition}}\;(\% ) = ({\text{A}}_{{{\text{control}}}} - {\text{A}}_{{{\text{sample}}}} )/{\text{A}}_{{{\text{control}}}} \times 100)$$

##### Ferric reducing antioxidant power (FRAP) assay

The FRAP method evaluating the ferric ion reducing capacity was determined as described by Benzie and Strain^31^ and Rufino et al.^32^. In a dark environment, an aliquot of 90 μL of each EO and their mixtures at 80 µg/mL in ethanol was mixed with 270 μL of distilled water and 2.5 mL of FRAP reagent (acetate buffer at 0.3 M—pH 3.6; TPTZ solution at 10 mM; ferric chloride solution at 20 mM). A control without the presence of EOs was also performed. The samples were left in a water bath for 30 min at 37 °C, followed by reading of absorbance values in a spectrophotometer (UV–VIS 1900i, Shimadzu, Tokyo, TKY, Japan) at 595 nm. The calibration curve was assembled based on TROLOX pure standard (from 160 to 800 µmol/L; R^2^ = 0.9995), and the results were expressed in μmol TROLOX equivalent/g. Both antioxidant assays were determined in triplicate.

#### MDs statistical analyses

The ANOVA with Tukey’s post-hoc test was used to certificate the significance of estimated coefficients . In addition, the desirability function (D) was used to obtain the optimal EO formulation for simultaneous better results in FRAP and DPPH assays. This optimized condition (critical points) was experimentally validated using Levene’s test and later applied in different concentrations in refrigerated stored trout fillets. The DoE was used for all these analyses (Statistica v.9.0 software, Stasoft, Tulsa, OK, USA) at a 95% confidence interval (*p* < 0.05).

### Application of optimal EO blend on rainbow trout fillets

#### Experimental design and application of the edible coating

Sixty fresh farmed rainbow trout (*Oncorhynchus mykiss*) fillets with skin were purchased in Rio de Janeiro, Brazil, and then were immediately transported in ice (0 °C) to the laboratory. The fillets (111.24 g ± 7.18 g each) were randomly divided into five treatments: control (fresh trout fillets with no added antioxidants); BHT (fresh trout fillets added of 100 ppm of butylhydroxytoluene); EO_100_ (fresh trout fillets added of 100 ppm of the optimized blend); EO_1000_ (fresh trout fillets added of 1000 ppm of the optimized blend); and EO_2000_ (fresh trout fillets added of 2000 ppm of the optimized blend). The active edible coatings were prepared by solubilizing the EO blends in a solution of tween 80 (0.8%, w/v) through Ultra Turrax 18 basic (IKA, Wilmington, NC, USA) (10,000 rpm for 5 min). The BHT was added directly (100 ppm, w/w) to the surface of the fillets, imitating industrial practice. The coating loaded with EO blends was evenly sprayed on the fish fillet surfaces (5 ± 0.5 mL) and dried on the dry flow cabin at room temperature for 2 min. Immediately after, they were individually air-packed in nylon/polyethylene bags and stored at 4 ± 1 °C. Samples were analyzed for total aerobic psychrotrophic count (TAPC), lipid and protein oxidation in triplicate, and instrumental color parameters and texture profile analysis in quadruplicate during nine days, based on the previous study of Monteiro et al.^33^. Each treatment was composed of 12 packages (three replicates × four days of storage; *n* = 3).

#### Determination of lipid oxidation

Lipid oxidation was evaluated by quantifying malondialdehyde (MDA) levels through thiobarbituric acid-reactive substances (TBARS) method (Yin et al.^34^) with slight adaptations^35^. Briefly, fillets were homogenized using an Ultra Turrax 18 basic (IKA, Wilmington, NC, USA) in trichloroacetic acid aqueous solution (TCA; 11%, w/v). Then, the content was centrifuged at 15,000 × *g* for 15 min at 4 °C, the supernatant was transferred to a test tube, where thiobarbituric acid aqueous solution (TBA; 20 mM, w/v) was added, the mixture was vortexed and incubated for 20 h in dark condition. The absorbance values were measured at 532 nm on a UV–VIS 1900i spectrophotometer (Shimadzu, Kyoto, Japan), and their conversion in mg MDA/kg fish muscle was obtained from a calibration curve (R^2^ = 0.999) built with seven MDA concentrations ranging from 1 to 500 µmol.

#### Determination of protein oxidation

Protein oxidation was quantified by the reaction between carbonyl groups and 2, 4 dinitrophenylhydrazine (DNPH) forming protein hydrazones, as described by Oliver et al. (1987) with modifications^36,37^. Briefly, a fillet aliquot was homogenized with potassium chloride solution (0.15 M; pH 7.4) using an Ultra Turrax 18 basic (IKA, Wilmington, NC, USA), followed by precipitation with trichloroacetic acid (TCA; 10%, w/v) and centrifugation (5000 × *g* for 5 min at 4 °C). Then, the precipitate was added of DNPH, incubated in dark conditions for 1 h, being vortexed every 15 min. After that, it was performed precipitation using TCA (20%, w/v), centrifugation (11,000 × *g* for 10 min at 4 °C), and washing three times with ethanol/ethyl acetate solution (1:1; v/v) with centrifugation at 15,000 × *g* for 10 min at 4 °C every wash. Then, the precipitate was dried and solubilized with 6 M guanidine hydrochloride in 20 mM sodium phosphate buffer (pH 6.5), followed by water bath at 50 °C for 15 min and centrifugation again at 11,000 × *g* for 10 min at 4 °C. For protein quantification, the absorbance values were read at 280 nm and converted in mg by a calibration curve built with five different concentrations of BSA (bovine serum albumin from 0.1 to 1.0 mg; R^2^ = 0.999). For carbonyl quantification, the absorbance values were measured at 370 nm using a UV–VIS 1900i spectrophotometer (Shimadzu, Kyoto, Japan), and the carbonyl content was expressed as nmol carbonyls/mg protein using an absorption coefficient of 21.0/mM/cm for protein hydrazones.

#### Total aerobic psychrotrophic count (TAPC)

For TAPC, 25 g of each trout fillet was transferred to sterile bags containing peptone water (225 mL; 0.1% w/v) and homogenized for 2 min (Stomacher, Millipore, Molsheim, France). Then, serial decimal dilutions were prepared in 0.1% peptone water (w/v) and spread in sterile Petri dishes containing Plate Count Agar (PCA; KASVI, Madrid, Spain). The plates were incubated at 7 °C for 10 days, and the colony forming units (CFU) were enumerated and expressed as log CFU/g.

#### Instrumental color measurements

After removing trout fillets from the packaging, lightness, redness and yellowness values were immediately measured through a portable spectrophotometer (Minolta CM-600D, Minolta Camera Co., Osaka, Japan) with an diameter aperture of 8 mm, standard observer (10°), and illuminant A. The color parameters were determined in four random places on the surface of each fillet.

#### Texture profile analysis (TPA)

Hardness, chewiness, cohesiveness, springiness, and resilience were measured with a Texture Analyser with a cylindrical P/36R probe (36 mm) (TA.XTplus, Stable Micro Systems, Surrey, UK). according to Bernardo et al.^38^.

### Statistical analysis

All analyses were performed with a 0.05 significance level using GraphPad Prism version 8.0.1 (GraphPad Sofware, San Diego, California, USA). The total amount of each dependent variable, except TAPC, produced over the storage was calculated (area under the curve—AUC); trapezoidal method). The differences among treatments (control, BHT, EO_100,_ EO_1000_, and EO_2000_) from AUC were identified by one-way ANOVA followed by post-hoc test with Tukey’s adjustment. For TAPC, the bacterial growth curves and their parameters (lag phase and µmax) were obtained (DMFit program, version 2.0, Norwich, UK), using the predictive primary model^39^, and through one-way ANOVA with Tukey post-hoc test (*p* < 0.05), the differences among treatments regarding lag phase and µmax were identified. Data of each parameter on each sampling day are shown in Supplementary Tables.

## Results and discussion

### Simplex-centroid design and antioxidant response

This study aimed to optimize the antioxidant effect through DPPH and FRAP values (dependent variables), and it is the first report with this approach, considering ORE, THY, and LG EO blends using the mixture design. The equations adjusted to the dependent variables are shown in Eqs. (3) and (4), where only terms with significant coefficients (*p* < 0.05) were considered. High or positive coefficient values mean independent variables (single EO or their mixtures) with increased antioxidant activity.

3 $$\begin{aligned} DPPH_{assay} & = 75.5455^{{{\mathbf{ORE}}}} \pm {1}{\text{.1727}} + 77.9028^{{{\mathbf{THY}}}} \pm {1}{\text{.1727}} + 96.0671^{{{\mathbf{LG}}}} \\ & \quad \pm {1}{\text{.1727}} - 24.4982^{{{\mathbf{ORE}} + {\mathbf{LG}}}} \pm {5}{\text{.2284}} \\ \end{aligned}$$

4 $$\begin{aligned} {\text{FRAP}}_{{{\text{assay}}}} & = 96.7068^{{{\mathbf{ORE}}}} \pm {3}{\text{.0982}} + 47.0992^{{{\mathbf{THY}}}} \pm {3}{\text{.0982}} + 17.0461^{{{\mathbf{LG}}}} \\ & \quad \pm {3}{\text{.0982}} - 42.0848^{{{\mathbf{ORE}} + {\mathbf{THY}}}} \pm {13}{\text{.8132}} \\ \end{aligned}$$

**Figure 1:**
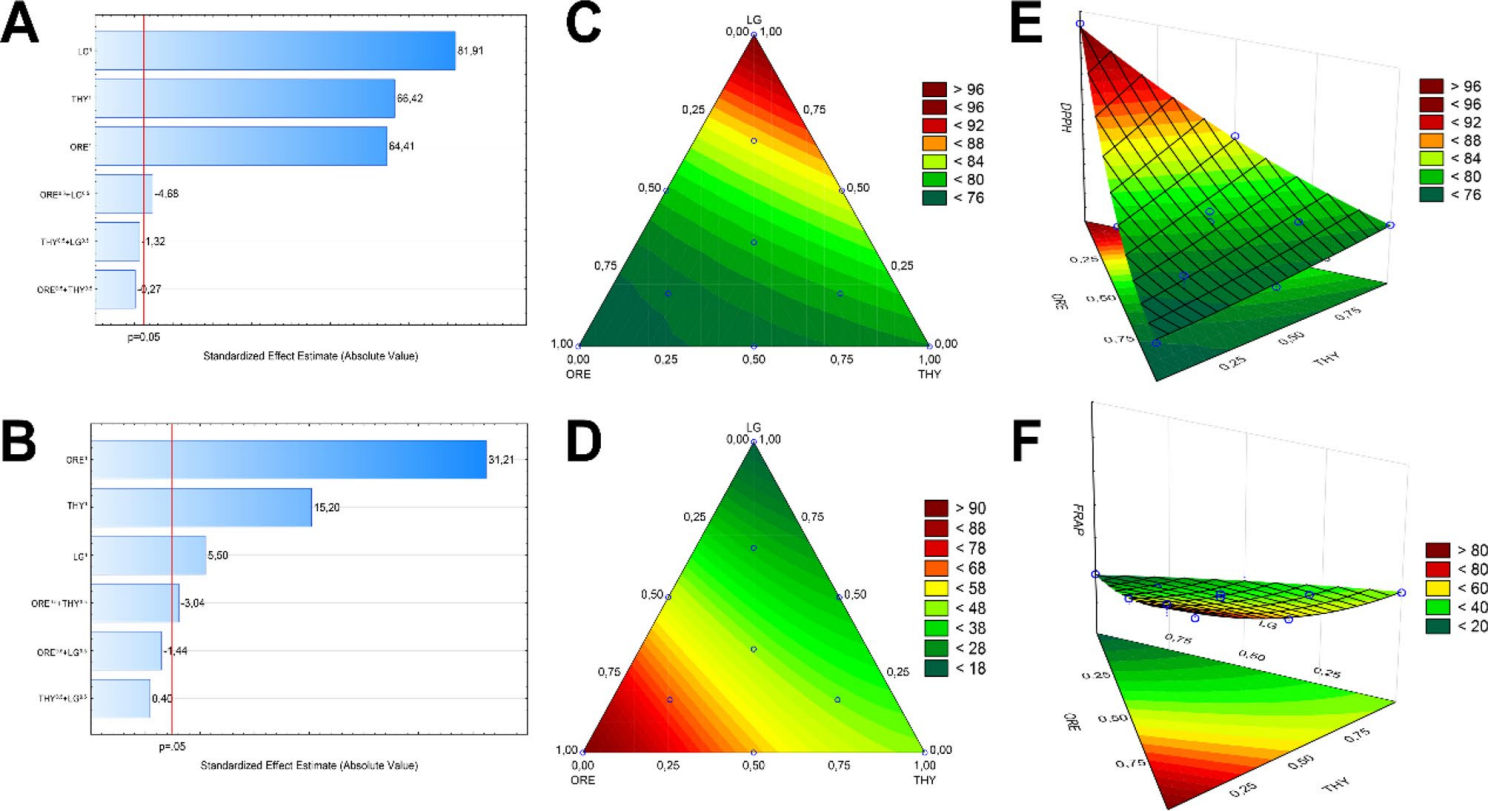
Pareto charts (**A**, **B**) indicating the most significant contribution of each EOs, 2D surface plots (**C**, **D**), and 3D surface plots (**E**, **F**) to show the action of essential oil blends with *Cymbopogon citratus* (LG), *Thymus vulgaris* (THY), and *Origanum vulgare* (ORE) on DPPH radical reducing (**A**, **C**, **E**) and ferric ion reducing (FRAP) (**B**, **D**, **F**). Results are expressed in percentage (%) of DPPH radical inhibition (**C**, **E**) and in μmol TROLOX equivalent/g (**D**, **F**).

Regarding single EOs, all of them showed significant effects regarding antioxidant activity, where the high coefficients were observed in the following decreasing order: LG > THY > ORE regarding DPPH activity (Eq. 3, Fig. 1C, E), and ORE > THY > LG for FRAP activity (Eq. 4, Fig. 1D, F). The antioxidant potential of these EOs has already been reported in the literature^21–23^ based on DPPH assay, where their activity was attributed to phenol compounds such as carvacrol and thymol (ORE and THY), and aldehydes such as neral and geranial (LG)^40–42^. Otherwise, the mixtures of EOs (proportion 50%:50%) did not show significant synergistic or additive interaction neither for DPPH nor FRAP activity.

The FRAP activity is solely based on the single electron transfer (SET) mechanism rather than DPPH activity, which looks at SET and hydrogen atom transfer (HAT) mechanisms^43,44^. In short, the antioxidant potential of EOs is mainly attributed to phenols due to their high reactivity, where peroxyl radicals are disposed through the hydrogen atom transfer^45^. On the other hand, neral and geranial show conjugated double bonds, which neutralize free radicals through the loss of allylic hydrogen atoms^46^. Nevertheless, EOs are complex mixtures constituted of other components with different polarities and chemical functions, such as hydrocarbons, alcohols, ketones, phenols, and ethers^16,47^.

**Figure 2:**
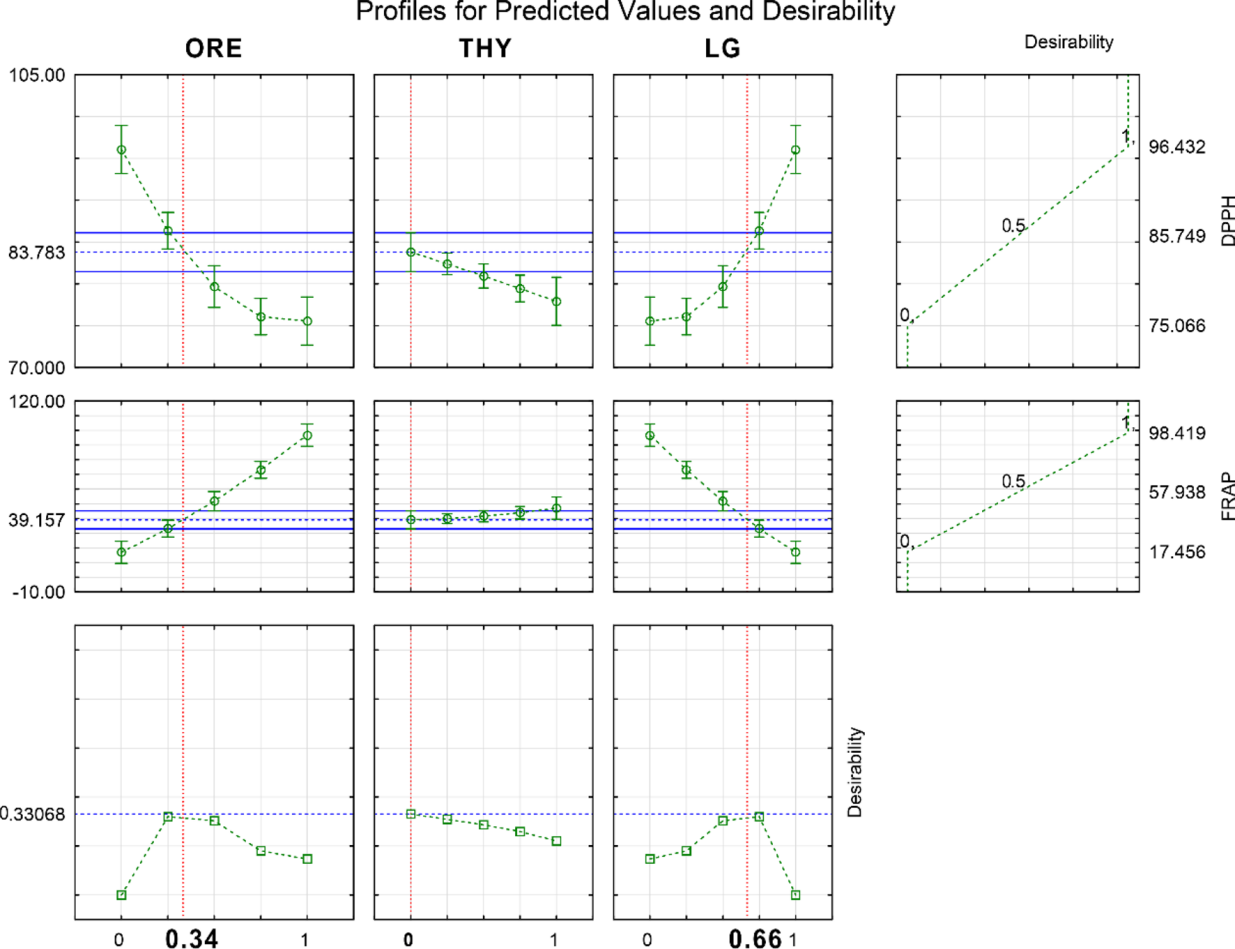
Desirability plot revealing the ideal ratios of *Cymbopogon citratus* (LG), *Thymus vulgaris* (THY), and *Origanum vulgare* (ORE) to the simultaneous DPPH radical reducing and ferric ion reducing (FRAP. Results are expressed in percentage (%) of DPPH radical inhibition and in μmol TROLOX equivalent/g for FRAP.

In our study, single EOs demonstrated antioxidant effectiveness via a specific mechanism (SET or HAT). Despite no significance for DPPH or FRAP activities separately, the EO blends (50%:50% ratio), mainly ORE and LG, exhibited a potential against oxidative degradation by concatenating mechanisms (Fig. 1A, B). It is crucial to prevent effectively complex reactions (e.g., lipid and protein oxidations) in a complex medium (e.g., food matrix), especially when high concentrations may not work. Therefore, the multiresponse optimization was used for EO blends aiming to achieve the highest antioxidant activity considering FRAP and DPPH activities simultaneously. The optimal blend obtained through the D function was 66% LG and 34% ORE, which would lead to 83.78% of DPPH inhibition and 39.15 μmol TROLOX equivalent/g (Fig. 2). This condition was validated experimentally in quadruplicate, resulting in 87.03% ± 0.77 of DPPH inhibition and 41.40 ± 0.54 μmol TROLOX equivalent/g. The Levene’s test showed an acceptable variance homogeneity since the *p*-values were 0.2804 for DPPH and 0.3505 for FRAP. For these reasons, the optimal EO blend was evaluated regarding its potential mitigate oxidative processes and to reduce bacterial growth in rainbow trout fillets stored for 9 days at 4 °C.

### Application of optimal EO blend on rainbow trout fillets over refrigerated storage for 9 days

#### Lipid and protein oxidation

**Figure 3:**
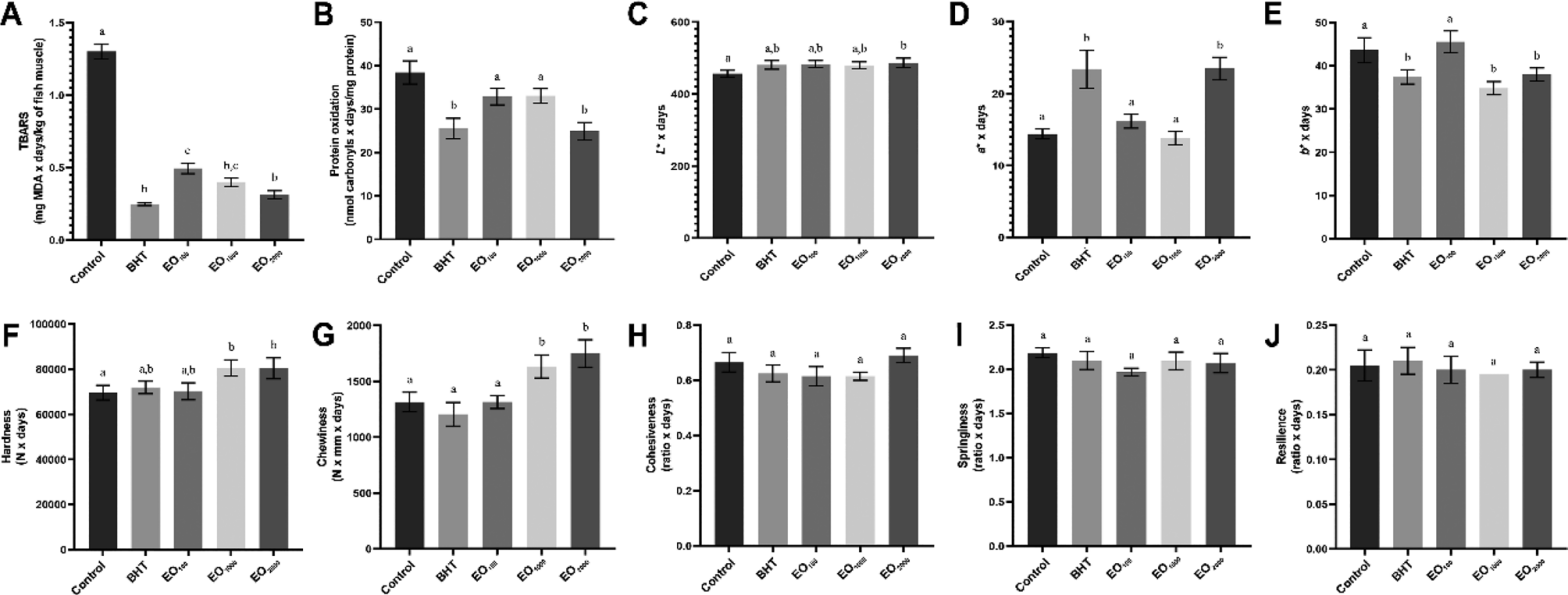
Lipid (**A**), protein oxidation (**B**), Lightness—L* (**C**), redness—a* (**D**), and yellowness—b* (**E**), hardness (**F**), chewiness (**G**), cohesiveness (**H**), springiness (**I**), and resilience (**J**) in rainbow trout (*Oncorhynchus mykiss*) fillets under refrigerated storage (4 °C) for nine days. Bars represent average ± standard deviation (*n* = 3). TBARS—thiobarbituric acid-reactive substances and MDA—malondialdehyde. Control (absence of antioxidant); BHT (100 ppm of butylhydroxytoluene); EO_100_ (100 ppm of the optimized blend); EO_1000_ (1000 ppm of the optimized blend); EO_2000_ (2000 ppm of the optimized blend). Different letters indicate significant differences (*p* < 0.05) among treatments over refrigerated storage.

The highest lipid oxidation during over refrigerated storage was observed in the control (*p* < 0.05; Fig. 3A). Regarding EO blends, EO_1000_ and EO_2000_ were similar to BHT, but no difference was also observed between EO_1000_ and EO_100_ (*p* > 0.05), and the last one exhibited higher lipid oxidation than BHT throughout storage (*p* < 0.05; Fig. 3A), indicating the potential of EO_2000_ in preventing lipid oxidation. Concerning carbonyl levels, the greater effect of EO_2000_ was more evident. The EO_2000_ was the only EO treatment that did not show a difference concerning BHT, while the other ones (EO_100_ and EO_1000_) demonstrated carbonyl content similar to control (*p* > 0.05; Fig. 3B), indicating that EO blend concentration equal to 2,000 ppm containing 66% LG and 34% ORE are as effective as BHT against protein oxidation.

Lipid and protein oxidations are initiated by some factors as light, reactive oxygen species (ROS), high temperature, pro-oxidant agents, and/or enzymes, and occur in a simultaneous way^6,48^. MDA from lipid oxidation binds to proteins (myoglobin and myofibrillar), increasing their susceptibility to oxidation reactions, while lipid oxidation is catalyzed by free iron released by protein oxidation^48,49^. Although there are some fluctuations, this may explain the similar effect of treatments on MDA and carbonyl levels during storage (Fig. 3A, B).

Studies evaluating the effect of EOs on lipid and protein oxidation of refrigerated stored fish are scarce and inexistent in the literature, respectively. Jouki et al.^50^ evaluated quince seed mucilage film (QSMF) loaded with oregano or thyme EOs at different concentrations (10,000, 15,000 and 20,000 ppm) in rainbow trout fillets stored under refrigeration. These authors reported that oregano EO at 20,000 ppm was the only effective treatment, reducing MDA values by almost 50% up to the 9th of storage. Mexis et al.^51^ observed that oregano EO at 4000 ppm was insufficient to reduce MDA values in refrigerated stored rainbow trout fillets for 9 days. In our study, concentrations 2 or 10 times smaller than those reported in the literature were able to prevent lipid and protein oxidation, controlling them by 76.13% and 35.13%, respectively, likewise BHT. This may be attributed to the HAT and SET mechanisms from the EO blend forming energetically stable species by supplying an H atom to the free radical and simultaneously transferring an electron^52,53^, which may enable greater control of the oxidation in trout fillets. Furthermore, the chelating capacity of ORE and LG, previously described in the literature^54,55^, may also be contributed to mitigate oxidative degradation in refrigerated stored trout fillets. Therefore, future studies are needed to more deeply understand the mechanisms of action of EOB in trout fillets.

### Total aerobic psychrotrophic count (TAPC)

The trout fillets showed good initial microbial quality with counts close to 3 log CFU/g on day 0^56^. The results of TAPC are shown in Table 3. No difference was observed in the growth rate (µmax) among treatments (*p* > 0.05). However, the lag phase was extended at the highest concentration of the EO blend (2000 ppm) when compared to the control and BHT (*p* < 0.05), increasing the shelf life by 13 h (Table 3). It is worth highlighting that we considered the limit of 7 log CFU/g^56^ as a microbiological criterion for the trout fillets’ shelf life. Furthermore, 13 h of shelf life extension for fish is a promising finding considering its high perishability and the corresponding effectiveness to BHT against oxidative degradation in a worst-scenario (dark muscle fish species and aerobic storage).

**Table 3:** Psychrotrophic growth parameters of rainbow trout (*Oncorhynchus mykiss*) fillets coated with optimized essential oil (EO) blend and stored under refrigeration (4 ± 1 °C) for nine days.

Treatments*	Lag phase^#^	µmax^#^	Shelf life^#^
Control	1.80 ± 0.58^a^	0.70 ± 0.06^a^	8.10
BHT	2.16 ± 0.35^a^	0.65 ± 0.02^a^	8.10
EO_100_	2.54 ± 0.30^a,b^	0.71 ± 0.05^a^	8.10
EO_1000_	2.52 ± 0.13^a,b^	0.66 ± 0.02^a^	8.28
EO_2000_	3.26 ± 0.30^b^	0.69 ± 0.03^a^	8.64

^#^Mean ± standard deviation (*n* = 3); Lag phase in days; µmax (exponential growth rate) in log colony-forming unit (CFU)/g/h; shelf life in days (≥ 7 log CFU/g; ICMSF, 2018). *Control (absence of antioxidant); BHT (100 ppm of butylhydroxytoluene); EO_100_ (100 ppm of the optimized blend); EO_1000_ (1000 ppm of the optimized blend); EO_2000_ (2000 ppm of the optimized blend).

Freshwater fish species are known to harbor Gram-negative aerobic and facultative anaerobic bacteria, wherein during the refrigerated aerobic storage, the *Pseudomonas* spp. became dominant (Gram and Huss^57^; Monteiro et al.^58^). The lag phase extension and the no effect on the growth rate found in this study can be explained by some factors. The antimicrobial combination of LG and ORE EOs has already been demonstrated previously against Gram-positive (*S. aureus*) and Gram-negative (*S.* Enteritidis and *E. coli*) bacteria^16^. However, the high amount of fat in trout muscle may have accelerated the EO blend penetration, favoring its effect on the lag phase rather than the growth rate. Besides, there are few studies on the antimicrobial activity of EOs against *Pseudomonas* spp. and the published data are controversial showing resistance and susceptibility of this genus to these oils (e.g., Lemongrass EO against *P. aeruginosa*)^59,60^. Furthermore, refrigerated storage under aerobic conditions is favorable to the growth of *Pseudomonas* spp.

This is the first study of the antibacterial potential of LG and ORE blend in trout fillet. In the study of Hosseini et al.^61^, the fish gelatin coating (FG) loaded with ORE at 1.2% (12,000 ppm) did not influence the shelf life of trout fillets stored under refrigeration for 8 days. Moreover, Raeisi et al.^62^ evaluated alginate or chitosan loaded with *Artemisia dracunculus*, *Mentha piperita*, or *Zataria multiflora* EO at 0.2% (2000 ppm) in refrigerated stored trout fillets for 12 days. These authors observed that all EO treatments, mainly chitosan loaded with *Zataria multiflora*, showed TAPC below 7 log CFU/g on day 12 when control exceeded the threshold, and it could extend shelf life in some hours.

#### Instrumental color parameters

Regarding L* values, the only difference found was a higher lightness in EO_2000_ than in control throughout the entire storage period (*p* < 0.05; Fig. 3C). The brightness of the EO blend on the fillet surface may have contributed to this finding. Concerning a* values, control, EO_100_, and EO_1000_ showed similar a* values throughout the refrigerated storage (*p* > 0.05), while BHT and EO_2000_ demonstrated the highest redness (*p* < 0.05; Fig. 3D) similarly. A decrease in a* values during the storage in dark fish species such as *Oncorhynchus mykiss* indicates metmyoglobin (MetMb) accumulation and, thus, meat discoloration^6,63^. This occurs due to myoglobin oxidation, where ferrous iron (Fe^2+^) is reduced to ferric iron (Fe^3+^) naturally during storage^63^. Therefore, higher a* values mean better prevention against discoloration, revealing the effectiveness of EO_2000_. These results corroborate and may be explained by our findings of protein oxidation.

For b* values, BHT, EO_1000,_ and EO_2000_ showed similarly the lowest yellowness (*p* < 0.05), and no difference was found between control and EO_100_ (*p* > 0.05; Fig. 3E) over the refrigerated storage. A similar pattern was observed in our results of lipid oxidation, which has been positively correlated with b* values throughout the refrigerated storage due to the production of yellow pigments during this oxidative process^6,64^. Previous studies have also reported the effect of EOs against lipid oxidation and its relationship with color parameters during the storage of trout fillets^65,66^.

#### Instrumental texture parameters

Overall, EO_1000_ and EO_2000_ showed the highest hardness and chewiness (*p* < 0.05), while control, BHT, and EO_100_ were similar throughout the storage (*p* > 0.05; Fig. 3F,G). Moreover, the other evaluated texture parameters were not affected (*p* > 0.05) by the presence of antioxidants (BHT and EO blends) during the refrigerated storage (Fig. 3H,I,J).

The hardness and chewiness were positively correlated with MDA levels and carbonyl content previously (Monteiro et al.^58^). In short, this effect is elucidated by the hydrophobic amino acids exposure of myofibrillar proteins caused by free radicals from lipid and protein oxidation, thereby increasing their exposure to endogenous and microbial proteases and decreasing hardness and chewiness^67,68^. Therefore, our results can be attributed to the antioxidant and antimicrobial activities of this blend (LG + ORE), decreasing oxidative reactions and availability of microbial proteases, thereby preserving more texture changes. Furthermore, BHT tended to preserve less hardness and chewiness than EO blends at 1000 and 2000 ppm (Fig. 3F,G), which can be attributed to this compound not having antimicrobial activity, allowing texture changes by microbial proteases. Jouki et al.^65^ observed an opposite behavior between microbiological growth and hardness in trout with QSM films loaded with ORE or THY. The stability observed in cohesiveness, springiness, and resilience values is well reported in refrigerated stored fish fillets, and it has been associated with muscle resistance in recovering its original parameters are related to form after deformation.

## Conclusion

Considering simultaneous FRAP and DPPH assays, the ideal EO blend was 66% of lemongrass and 34% of oregano. This blend at 2000 ppm was equally effective to BHT against lipid and protein oxidation/discoloration and better than this synthetic antioxidant for preserving texture changes and enhancing the shelf life, revealing a promising natural alternative to improve the quality of rainbow trout fillets under refrigerated storage. Moreover, the multiresponse optimization showed to be a strong ally in enabling the use of these EOs by food industries. Nevertheless, as this is the first study optimizing ORE, THY, and LG EO blend and further applying it in refrigerated stored trout fillets, studies towards sensory evaluation and combining this EO blend with other carriers (e.g., coatings, films, emulsions, nanocapsules, among others) are encouraged.

### Supplementary Information

Supplementary Tables.

## Data Availability

The datasets generated and/or analyzed during the current study are available from the corresponding author on reasonable request.
