# Supplementary material for: Oil-in-water emulsion loaded with optimized antioxidant blend improved the shelf-life of trout (Oncorhynchus mykiss) fillets: a study with simplex-centroid design
Source: Sci Rep. 2024 Feb 27;14:4810. doi: 10.1038/s41598-024-55308-x (PMC10899203; doi:10.1038/s41598-024-55308-x)
Supplement: Supplementary file 1 — Supplementary Tables. [file 41598_2024_55308_MOESM1_ESM.docx]

| Treatments | Days of storage^*^ | | | |
| --- | --- | --- | --- | --- |
|  | 0 | 3 | 6 | 9 |
| Control | 0.174 ± 0.010 | 0.023 ± 0.003 | 0.283 ± 0.017 | 0.085 ± 0.011 |
| BHT | 0.004 ± 0.000 | 0.011 ± 0.001 | 0.031 ± 0.003 | 0.074 ± 0.002 |
| EO_100_ | 0.069 ± 0.004 | 0.022 ± 0.003 | 0.017 ± 0.002 | 0.183 ± 0.034 |
| EO_1000_ | 0.010 ± 0.002 | 0.025 ± 0.003 | 0.022 ± 0.001 | 0.164 ± 0.022 |
| EO_2000_ | 0.016 ± 0.001 | 0.017 ± 0.002 | 0.052 ± 0.008 | 0.052 ± 0.010 |

**Supplementary Table 1.** Lipid oxidation (TBARS - thiobarbituric acid-reactive substances) in rainbow trout (*Oncorhynchus mykiss*) fillets coated with optimized essential oil (EO) blend and stored at 4 ± 1°C for 9 days..

**^*^**Results are expressed as mean ± standard deviation (*n* = 3) in mg malondialdehyde/kg fish muscle. Control (absence of antioxidant); BHT (100 ppm of butylhydroxytoluene); EO_100_ (100 ppm of the optimized blend); EO_1000_ (1,000 ppm of the optimized blend); EO_2000_ (2,000 ppm of the optimized blend).

**Supplementary Table 2.** Protein oxidation in rainbow trout (*Oncorhynchus mykiss*) fillets coated with optimized essential oil (EO) blend and stored at 4 ± 1°C for 9 days.

| Treatments | days of storage^*^ | | | |
| --- | --- | --- | --- | --- |
|  | 0 | 3 | 6 | 9 |
| Control | 5.130 ± 0.729 | 2.566 ± 0.265 | 5.468 ± 0.624 | 4.411 ± 0.506 |
| BHT | 2.633 ± 0.214 | 2.715 ± 0.596 | 2.529 ± 0.549 | 3.900 ± 0.163 |
| EO_100_ | 2.744 ± 0.516 | 4.316 ± 0.339 | 3.045 ± 0.455 | 4.432 ± 0.099 |
| EO_1000_ | 2.700 ± 0.115 | 3.507 ± 0.378 | 4.075 ± 0.248 | 4.165 ± 0.477 |
| EO_2000_ | 2.112 ± 0.100 | 2.466 ± 0.416 | 2.892 ± 0.419 | 3.782 ± 0.480 |

**^*^**Results are expressed as mean ± standard deviation (*n* = 3) in nmol carbonyls/mg protein. Control (absence of antioxidant); BHT (100 ppm of butylhydroxytoluene); EO_100_ (100 ppm of the optimized blend); EO_1000_ (1,000 ppm of the optimized blend); EO_2000_ (2,000 ppm of the optimized blend).

**Supplementary Table 3.** Total aerobic psychrotrophic count (TAPC) in rainbow trout (*Oncorhynchus mykiss*) fillets coated with optimized essential oil (EO) blend and stored at 4 ± 1°C for 9 days.

| Treatments | days of storage^*^ | | | |
| --- | --- | --- | --- | --- |
|  | 0 | 3 | 6 | 9 |
| Control | 3.02 ± 0.00 | 3.70 ± 0.20 | 6.01 ± 0.15 | 7.58 ± 0.01 |
| BHT | 3.29 ± 0.10 | 3.67 ± 0.06 | 6.02 ± 0.00 | 7.46 ± 0.02 |
| EO_100_ | 3.19 ± 0.06 | 3.33 ± 0.22 | 5.90 ± 0.04 | 7.52 ± 0.02 |
| EO_1000_ | 3.27 ± 0.12 | 3.54 ± 0.16 | 5.72 ± 0.12 | 7.36 ± 0.17 |
| EO_2000_ | 3.34 ± 0.13 | 3.39 ± 0.00 | 5.28 ± 0.11 | 7.18 ± 0.03 |

**^*^**Results are expressed as mean ± standard deviation (*n* = 3) in log CFU/g. Control (absence of antioxidant); BHT (100 ppm of butylhydroxytoluene); EO_100_ (100 ppm of the optimized blend); EO_1000_ (1,000 ppm of the optimized blend); EO_2000_ (2,000 ppm of the optimized blend).

**Supplementary Table 4.** *L** (lightness), *a** (redness), and *b** (yellowness) values in rainbow trout (*Oncorhynchus mykiss*) fillets coated with optimized essential oil (EO) blend and stored at 4 ± 1°C for 9 days.

|  | Treatments | Days of storage^*^ | | | |
| --- | --- | --- | --- | --- | --- |
|  |  | 0 | 3 | 6 | 9 |
| *L** | Control | 50.22 ± 1.83 | 50.32 ± 2.34 | 51.49 ± 0.58 | 50.41 ± 2.02 |
|  | BHT | 53.18 ± 0.90 | 54.04 ± 2.43 | 52.82 ± 2.97 | 53.62 ± 0.79 |
|  | EO_100_ | 53.50 ± 1.72 | 52.69 ± 0.94 | 54.40 ± 2.25 | 54.34 ± 2.68 |
|  | EO_1000_ | 54.75 ± 1.98 | 52.58 ± 2.73 | 53.09 ± 1.27 | 53.64 ± 0.52 |
|  | EO_2000_ | 51.00 ± 2.02 | 55.89 ± 1.16 | 53.96 ± 2.81 | 53.71 ± 4.52 |
|  |  |  |  |  |  |
| *a** | Control | 1.30 ± 0.27 | 2.19 ± 0.05 | 1.76 ± 0.12 | 0.40 ± 0.01 |
|  | BHT | 2.75 ± 0.36 | 1.85 ± 0.50 | 4.00 ± 0.74 | 1.15 ± 0.14 |
|  | EO_100_ | 1.74 ± 0.17 | 2.47 ± 0.25 | 1.49 ± 0.11 | 1.11 ± 0.20 |
|  | EO_1000_ | 1.38 ± 0.07 | 1.55 ± 0.30 | 0.89 ± 0.06 | 2.94 ± 0.00 |
|  | EO_2000_ | 2.12 ± 0.13 | 2.77 ± 0.48 | 1.75 ± 0.18 | 4.51 ± 0.00 |
|  |  |  |  |  |  |
| *b** | Control | 4.70 ± 0.32 | 3.83 ± 0.60 | 6.46 ± 0.79 | 3.80 ± 0.33 |
|  | BHT | 2.58 ± 0.31 | 4.16 ± 0.38 | 4.75 ± 0.28 | 4.54 ± 0.31 |
|  | EO_100_ | 4.75 ± 0.21 | 5.46 ± 0.63 | 5.60 ± 0.56 | 3.49 ± 0.20 |
|  | EO_1000_ | 2.38 ± 0.13 | 4.64 ± 0.21 | 4.16 ± 0.49 | 3.23 ± 0.12 |
|  | EO_2000_ | 2.90 ± 0.42 | 5.66 ± 0.35 | 4.20 ± 0.12 | 2.69 ± 0.23 |

**^*^**Results are expressed as mean ± standard deviation (*n* = 3); Control (absence of antioxidant); BHT (100 ppm of butylhydroxytoluene); EO_100_ (100 ppm of the optimized blend); EO_1000_ (1,000 ppm of the optimized blend); EO_2000_ (2,000 ppm of the optimized blend).

**Supplementary Table 5.** Texture profile of rainbow trout (*Oncorhynchus mykiss*) fillets coated with optimized essential oil (EO) blend and stored at 4 ± 1°C for 9 days.

|  | Treatments | Days of storage ^*^ | | | |
| --- | --- | --- | --- | --- | --- |
|  |  | 0 | 3 | 6 | 9 |
| Hardness (N) | Control | 8206.13 ± 905.01 | 7762.37 ± 599.43 | 6746.10 ± 415.23 | 9145.05 ± 868.47 |
|  | BHT | 7740.82 ± 1069.90 | 7732.96 ± 433.12 | 8016.25 ± 217.67 | 8666.20 ± 468.80 |
|  | EO_100_ | 7237.23 ± 439.99 | 7414.73 ± 383.03 | 8503.19 ± 948.08 | 7708.55 ± 915.21 |
|  | EO_1000_ | 7762.00 ± 689.29 | 9076.07 ± 840.96 | 9652.07 ± 532.86 | 8445.19 ± 879.12 |
|  | EO_2000_ | 7947.44 ± 367.08 | 8979.25 ± 588.59 | 9220.71 ± 1136.02 | 9201.49 ± 995.56 |
|  |  |  |  |  |  |
| Chewiness (N x mm) | Control | 130.23 ± 18.95 | 137.34 ± 13.03 | 122.88 ± 9.14 | 225.62 ± 29.41 |
|  | BHT | 113.72 ± 9.11 | 150.19 ± 27.66 | 87.89 ± 4.52 | 213.65 ± 28.19 |
|  | EO_100_ | 96.17 ± 0.70 | 127.98 ± 3.95 | 178.94 ± 15.26 | 166.61 ± 17.21 |
|  | EO_1000_ | 118.10 ± 8.94 | 167.14 ± 7.90 | 196.20 ± 15.86 | 243.91 ± 40.87 |
|  | EO_2000_ | 147.11 ± 2.76 | 199.83 ± 27.38 | 136.00 ± 5.60 | 347.13 ± 47.26 |
|  |  |  |  |  |  |
| Cohesiveness (ratio) | Control | 0.06 ± 0.00 | 0.08 ± 0.01 | 0.07 ± 0.01 | 0.10 ± 0.01 |
|  | BHT | 0.06 ± 0.01 | 0.07 ± 0.01 | 0.06 ± 0.01 | 0.08 ± 0.00 |
|  | EO_100_ | 0.06 ± 0.00 | 0.06 ± 0.00 | 0.07 ± 0.01 | 0.09 ± 0.01 |
|  | EO_1000_ | 0.06 ± 0.00 | 0.07 ± 0.00 | 0.06 ± 0.00 | 0.08 ± 0.00 |
|  | EO_2000_ | 0.07 ± 0.01 | 0.08 ± 0.01 | 0.06 ± 0.00 | 0.10 ± 0.00 |
|  |  |  |  |  |  |
| Springiness (ratio) | Control | 0.21 ± 0.01 | 0.27 ± 0.02 | 0.22 ± 0.01 | 0.27 ± 0.01 |
|  | BHT | 0.21 ± 0.02 | 0.24 ± 0.02 | 0.22 ± 0.01 | 0.27 ± 0.02 |
|  | EO_100_ | 0.20 ± 0.02 | 0.23 ± 0.00 | 0.21 ± 0.00 | 0.24 ± 0.01 |
|  | EO_1000_ | 0.20 ± 0.01 | 0.26 ± 0.01 | 0.21 ± 0.02 | 0.25 ± 0.02 |
|  | EO_2000_ | 0.23 ± 0.01 | 0.24 ± 0.02 | 0.20 ± 0.02 | 0.27 ± 0.03 |
|  |  |  |  |  |  |
| Resilience (ratio) | Control | 0.02 ± 0.00 | 0.02 ± 0.00 | 0.02 ± 0.00 | 0.03 ± 0.00 |
|  | BHT | 0.02 ± 0.00 | 0.02 ± 0.00 | 0.02 ± 0.00 | 0.03 ± 0.00 |
|  | EO_100_ | 0.02 ± 0.00 | 0.02 ± 0.00 | 0.02 ± 0.00 | 0.03 ± 0.00 |
|  | EO_1000_ | 0.02 ± 0.00 | 0.02 ± 0.00 | 0.02 ± 0.00 | 0.03 ± 0.00 |
|  | EO_2000_ | 0.02 ± 0.00 | 0.02 ± 0.00 | 0.02 ± 0.00 | 0.03 ± 0.00 |

**^*^**Results are expressed as mean ± standard deviation (*n* = 3); Control (absence of antioxidant); BHT (100 ppm of butylhydroxytoluene); EO_100_ (100 ppm of the optimized blend); EO_1000_ (1,000 ppm of the optimized blend); EO_2000_ (2,000 ppm of the optimized blend).
